# Lactate-Albumin Ratio: A Novel Predictor of Noninvasive Mechanical Ventilation Failure in Acute Hypercapnic Respiratory Failure

**DOI:** 10.7759/cureus.83314

**Published:** 2025-05-01

**Authors:** Umut Kasapoglu, Betul Gunay, Cansu Unlu, Caner Cinar, Huseyin Arikan, Erdem Yalcinkaya, Zeynep Mercanci, Derya Kocakaya, Sehnaz Olgun Yıldızeli, Emel Eryuksel, Sait Karakurt

**Affiliations:** 1 Department of Pulmonary and Critical Care Medicine, Marmara University School of Medicine, Istanbul, TUR

**Keywords:** albumin, failure, lactate, lactate/albumin ratio, noninvasive mechanical ventilation, predictor

## Abstract

Background: Acute respiratory failure (ARF) is a leading cause of patient admissions to intensive care units (ICUs). Noninvasive mechanical ventilation (NIMV) is a cornerstone in the management of ARF, particularly in hypercapnic respiratory failure. However, NIMV failure can occur due to various factors, including the underlying cause of ARF. While the lactate/albumin ratio (LAR) has been studied as a prognostic biomarker in various critical conditions, this study is the first to specifically evaluate its role in predicting NIMV failure in patients with acute hypercapnic respiratory failure due to COPD exacerbation.

Methods: This retrospective study included 116 patients admitted to the Level 3 medical ICU at Marmara University Pendik Training and Research Hospital between January 1, 2019, and January 1, 2023. All patients were admitted for acute hypercapnic respiratory failure secondary to an exacerbation of chronic obstructive pulmonary disease (COPD) and were treated with NIMV. Only patients who were followed in the ICU for more than 24 hours were included.

Results: Of the 116 patients, 72 (62.6%) were male, with a mean age of 70±10 years. NIMV failure occurred in 37% of the cases. There were no statistically significant differences between the NIMV success and failure groups in terms of mean age, Acute Physiology and Chronic Health Evaluation-II (APACHE-II) scores, or heart rate, acidosis, consciousness, oxygenation, and respiratory rate (HACOR) scores at ICU admission (p=0.746, p=0.565, p=0.403, respectively). However, the NIMV failure group had significantly higher Sequential Organ Failure Assessment (SOFA) scores at ICU admission (p=0.002). Furthermore, the NIMV failure group exhibited lower serum albumin levels, higher serum lactate levels, and elevated LAR values (p=0.029, p=0.009, p=0.004, respectively). LAR (AUC: 0.718, p<0.001) proved to be a superior predictor of NIMV failure compared to serum albumin (AUC: 0.603, p=0.066) and blood lactate (AUC: 0.684, p=0.001) levels alone. A LAR cut-off value of 0.605 was identified as predictive of NIMV failure. Regression analysis indicated that each unit increase in LAR was associated with a 5.582-fold increase in the risk of intubation (p=0.015).

Conclusions: LAR was identified as a significant independent risk factor for NIMV failure. Early monitoring of LAR during NIMV application in 116 patients with acute COPD exacerbation may provide clinicians with valuable insight to guide timely intubation decisions, potentially improving patient outcomes.

## Introduction

Over the past two decades, the use of noninvasive mechanical ventilation (NIMV) has significantly advanced, becoming a cornerstone treatment for acute respiratory failure (ARF) across various clinical settings. Its implementation in managing acute hypercapnic respiratory failure due to chronic obstructive pulmonary disease (COPD) has notably reduced intubation rates, mortality, complication rates, and hospital stay durations [1-3].

Clinically, NIMV failure is defined as the necessity for endotracheal intubation (ETI) or patient death despite NIMV support [4]. Timely identification of patients likely to fail NIMV is crucial since delays in intubation correlate with increased mortality. Despite protocol adherence, NIMV failure rates remain variable, ranging widely from 5% to 60%, influenced by patient-specific factors, severity of illness, and intervention timing. However, unknown or insufficiently understood risk factors further complicate the predictability of NIMV outcomes, underscoring the necessity for continued research [3,5].

Early recognition of patients unlikely to benefit from NIMV, thus requiring timely intubation, remains a critical clinical challenge. Reliable prognostic biomarkers could significantly enhance patient stratification, allowing for individualized management decisions and potentially reducing invasive ventilation needs. Identifying novel and accurate predictors of NIMV failure could therefore improve both immediate and long-term patient outcomes [3,4,6].

Recent studies have identified the lactate/albumin ratio (LAR) as a robust prognostic biomarker in critically ill patients, particularly in conditions like septic shock, heart failure, and cardiac arrest. Compared to isolated lactate or albumin measurements, LAR offers superior prognostic accuracy by simultaneously reflecting metabolic stress and nutritional status. Consequently, LAR holds promise for enhanced risk stratification and dynamic monitoring of patient response to therapy in intensive care units (ICUs) [7-12].

Nevertheless, the specific utility of LAR in predicting NIMV failure among patients with COPD-related acute hypercapnic respiratory failure remains uncertain. This study aims to address this gap by evaluating the prognostic value of LAR for predicting NIMV failure in this patient population.

## Materials and methods

Study design and patient population

The retrospective cohort study was conducted in the Level 3 medical ICU of Marmara University Hospital between January 2019 and January 2023. A total of 116 patients who were followed up in the medical ICU for more than 24 hours and underwent NIMV due to hypercapnic respiratory failure induced by acute exacerbations of COPD were included in the study. The study was approved by the Ethics Committee of Marmara University School of Medicine (approval number: 09.2023.716, date: 05.05.2023). Due to the retrospective nature of the study, the requirement for informed patient consent was waived.

Inclusion and exclusion criteria

The inclusion criteria for the study were as follows: a previous diagnosis of COPD; hypercapnic ARF with pH <7.35 and PaCO₂ >45 mmHg, with or without hypoxemia (PaO₂/FiO₂ <300); ability to cough effectively and expectorate secretions; hemodynamic stability; and the ability to tolerate and comply with a face or mask interface for NIMV.

The exclusion criteria for the study were as follows: a diagnosis other than COPD; hypoxemic ARF with PaO₂/FiO₂ <300 and PaCO₂ <45 mmHg; transfer to another ICU after initiation of NIMV; ICU stay of less than 24 hours; death within 24 hours of admission; inability to protect the airway; and non-cooperation with NIMV application.

Initiation of NIMV therapy

NIMV was initiated in the presence of at least one of the following clinical criteria: respiratory acidosis (pH <7.35, pCO₂ >45 mmHg), severe dyspnea with clinical signs of respiratory muscle fatigue, including the use of accessory respiratory muscles, paradoxical abdominal motion, or intercostal muscle retraction [1,2,13]. Despite optimized medical treatment, patients who continued to experience respiratory acidosis and hypercapnia were also considered candidates for NIMV initiation.

Equipment and setup: Hospital-dedicated NIMVs (Respironics V60 Ventilator by Philips Respironics, United States) with a single-tube circuit should be utilized. To enhance patient adherence to treatment, a full-face mask is typically recommended.

Initial settings: Expiratory positive airway pressure (EPAP) was initially set at 4 cmH₂O. If necessary, EPAP was increased in increments of 2-5 cmH₂O to achieve oxygenation targets of arterial oxygen pressure (PaO₂) ≥60 mmHg or oxygen saturation (SpO₂) ≥90%. Inspiratory positive airway pressure (IPAP) was adjusted to deliver a tidal volume >5 mL/kg, provided the patient tolerated it [13].

Duration and monitoring: NIMV is applied intermittently for periods ranging from one to four hours. Arterial blood gas samples are obtained within one to two hours of initiation to evaluate the effectiveness of ventilation and oxygenation.

Criteria for NIMV failure

NIMV failure is defined as the need for invasive mechanical ventilation or the occurrence of death in a patient receiving NIMV [3].

Data collection

We collected and analyzed the following data: demographic and clinical characteristics of all patients; ICU admission scores from the Acute Physiology and Chronic Health Evaluation II (APACHE II), Sequential Organ Failure Assessment (SOFA), and the Heart Rate, Acidosis, Consciousness, Oxygenation, and Respiratory Rate (HACOR) score; vital signs upon ICU admission; laboratory parameters; and arterial blood gas analysis at ICU admission. Additional outcomes included the need for invasive mechanical ventilation, ICU length of stay, and 30-day survival status. All blood samples, including lactate and albumin levels, were collected simultaneously upon ICU admission to ensure consistency in the evaluation of biochemical parameters. The recorded data were compared between the NIMV failure and NIMV success groups, and risk factors for NIMV failure in the ICU were analyzed. There were no missing data among the variables included in the analysis.

Measurement of outcome

We monitored all critically ill COPD patients throughout their ICU stays or until death in the ICU. Additionally, data regarding the need for invasive mechanical ventilation and 30-day mortality were collected for all patients using the hospital's electronic medical record system.

Statistical analysis

The patients’ data were obtained by scanning the hospital medical record system. Subsequently, the data included in the study were analyzed using IBM SPSS V25 (Statistical Package for Social Sciences; SPSS Inc., Chicago, IL). All results were analyzed with a confidence interval of 95% and a significance level of P<0.05. The homogeneity and distribution of the variables were assessed using Skewness-Kurtosis. Frequencies and percentages were used for categorical data, mean values ± standard deviation for parametric variables, and median (min-max) values for non-parametric variables. The chi-squared test was employed to compare categorical variables. The Independent Samples T-Test was used to analyze parametric variables in two independent groups, while the Mann-Whitney U Test was employed for non-parametric variables. Cut-off values for predicting NIMV failure were examined using ROC analysis. Independent risk factors affecting the success of NIMV were examined using binary logistic regression analysis.

## Results

Among the study population, 72 patients (62.6%) were male, with a mean age of 70±10 years. Comorbidities were present in 85 patients (73.3%), with hypertension being the most prevalent. The incidence of NIMV failure was 37.2%. Patients were stratified into two groups: Group 1, comprising those who experienced NIMV failure (n=43, 37%), and Group 2, consisting of those with successful NIMV outcomes (n=73, 63%). There were no statistically significant differences in demographic characteristics between the groups (Table 1).

**Table 1:** Comparison of demographics and comorbidities in NIMV success versus failure *Independent Samples T-Test **Chi-squared test NIMV: noninvasive mechanical ventilation, χ² value: chi-squared test value, df: degrees of freedom, HT: hypertension, CHD: coronary heart diseases, DM: diabetes mellitus, CKD: chronic kidney diseases

		NIMV success (n=73)	NIMV failure (n=43)	χ^² ^value	df	Cramer’s value	p-value
Mean age, years		70±11	70±10				0.746^*^
Gender, n (%)	Male	43 (58.9%)	30 (69.7%)	1.369	1	0.109	0.244^**^
Presence of at least one comorbidity	Yes	52 (71.2%)	33 (76.7%)	0.420	1	0.060	0.517^**^
Coexisting comorbidities, n (%)	HT	39 (53.4%)	29 (67.4%)	2.192	1	0.137	0.141^**^
	CHD	16 (21.9%)	11 (25.5%)	0.203	1	0.042	0.652^**^
	CHF	12 (16.4%)	8 (18.8%)	0.089	1	0.028	0.766^**^
	DM	24 (32.8%)	14 (32.5)	0.001	1	0.003	0.972^**^
	CKD	14 (19.1%)	5 (11.6%)	1.126	1	0.099	0.293^**^
	Malignancy	5 (6.8%)	5 (11.6%)	0.784	1	0.082	0.381^**^

There were no significant differences between the two groups in terms of vital signs. Additionally, no statistically significant differences were observed between the two groups in terms of APACHE-II and HACOR scores at the time of ICU admission (p=0.565, p=0.403, respectively). However, the group with NIMV failure demonstrated significantly lower Glasgow Coma Scale (GCS) scores compared to the NIMV success group (p<0.005), indicating a higher degree of impaired consciousness. In terms of disease severity, the NIMV failure group exhibited significantly higher SOFA scores at the time of ICU admission (p=0.004), suggesting greater overall organ dysfunction and illness severity compared to the NIMV success group (Table 2).

**Table 2:** Comparison of physical examination findings and disease severity between NIMV success and failure groups *Independent Samples T-Test **Chi-squared test NIMV: noninvasive mechanical ventilation, SBP: systolic blood pressure, DAB: diastolic blood pressure, RR: respiratory rate, HR: heart rate, GCS: Glasgow Coma Scale, APACHE-II: Acute Physiology and Chronic Health Evaluation-II, SOFA: Sequential Organ Failure Assessment, HACOR: Heart Rate, Acidosis, Consciousness, Oxygenation, and Respiratory Rate, SD: standard deviation

	NIMV success (n=73)	NIMV failure (n=43)	p-value
SBP, mmHg, mean±SD	126±21	114±28	0.056^*^
DBP, mmHg, mean±SD	70±18	64±14	0.053^*^
RR, bpm, mean±SD	29±5	27±8	0.095^*^
HR, bpm, mean±SD	87±17	88±22	0.878^*^
GCS, median (min-max)	15 (12-15)	14 (10-15)	<0.001^**^
APACHE-II score, mean±SD	17±3	17±3	0.562^*^
SOFA score, mean±SD	3±2	5±2	0.004^*^
HACOR score, mean±SD	6±2	7±2	0.403^*^

In our analysis, we compared the laboratory data of patients at the time of ICU admission. Patients in the NIMV failure group were found to have significantly different laboratory parameters compared to those in the NIMV success group. Specifically, the NIMV failure group presented with a lower serum pH level (p=0.002), higher pCO₂ levels (p=0.011), elevated blood lactate levels (p=0.009), and increased LAR values (p=0.004) (Table 3).

**Table 3:** Baseline laboratory findings of the patients *Independent Samples T-Test **Chi-squared test Hgb: hemoglobin, Hct: hematocrit, Wbc: white blood cells, Neu: neutrophils, lymph: lymphocytes, Plt: platelets, AST: aspartate aminotransferase, ALT: alanine aminotransferase, CK: creatine kinase, LDH: lactate dehydrogenase, T. bil: total bilirubin, NT-proBNP: N-terminal prohormone of brain natriuretic peptide, INR: international normalized ratio, CRP: C-reactive protein, SD: standard deviation, med: median, LAR: lactate albumin ratio

		NIMV success (n=73)	NIMV failure (n=43)	p-value
Hemogram	Hgb, g/dL, mean±SD	11.6±2.62	11.41±2.71	0.715^*^
	Hct, %, mean±SD	36.36±7.95	35.63±8.04	0.633^*^
	Wbc, 10^3^/μL, mean±SD	11.40±4.90	11.71±6.70	0.769^*^
	Neu, 10^3^/μL, mean±SD	10.09±4.76	10.02±5.95	0.950^*^
	Lymph, 10^3^/μL, mean±SD	0.70±0.53	0.89±0.76	0.124^*^
	Plt, 10^3^/μL, mean±SD	249±126	238±90	0.633^*^
Biochemical parameters	BUN, mg/dL, mean±SD	30±16	36±25	0.113^*^
	Creatinin, mg/dL, mean±SD	1.14±0.97	1.4±1.14	0.205^*^
	AST, U/L, mean±SD	42±95	56±94	0.473^*^
	ALT, U/L, mean±SD	33±47	44±59	0.304^*^
	LDH, IU/L, mean±SD	334±195	515±973	0.257^*^
	T. bil, mg/dL, med. (min-max)	0.57 (0.10-3.17)	0.58 (0.12-4.22)	0.980^**^
	Albumin, g/dL, mean±SD	3.45±0.5	3.21±0.58	0.029^*^
	NT-proBNP, pg/mL, mean±SD	5381±8689	6148±8226	0.638^*^
	CRP, mg/dL, mean±SD	48±60	84±96	0.023^*^
	LAR, mean±SD	0.46±0.33	0.71±0.46	0.004^*^
Arterial blood gas analysis	pH, mean±SD	7.28±0.06	7.23±0.09	0.002^*^
	PO_2_, mmHg, mean±SD	95±48	102±52	0.430^*^
	PCO_2_, mmHg, mean±SD	70±12	79±24	0.011^*^
	HCO_3_, mEq/L, mean±SD	29.39±5.98	29.04±7.35	0.778^*^
	Lactate, mmol/L, mean±SD	1.55±1.09	2.23±1.34	0.009^*^
	SaO_2_, %, mean±SD	94±18	96±16	0.396^*^
	Horowitz index, mean±SD	158±81	171±86	0.411^*^

**Figure 1:**
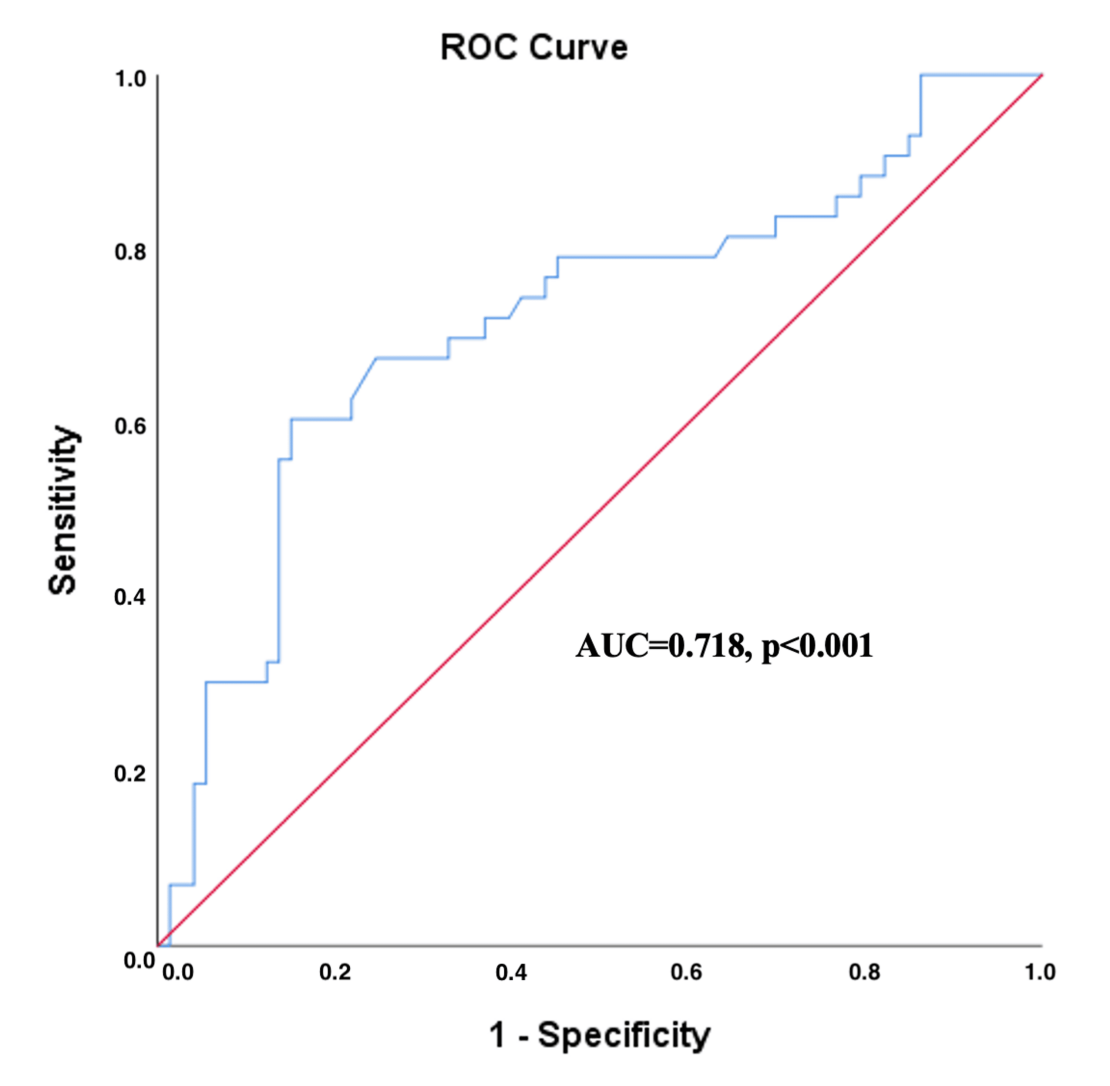
The ROC curve presented in this figure demonstrates the diagnostic accuracy of the LAR for predicting NIMV failure in patients with acute hypercapnic respiratory failure. The AUC is 0.718, with a statistically significant p-value of <0.001 NIMV: noninvasive mechanical ventilation, LAR: lactate albumin ratio

We evaluated the performance of LAR, serum albumin, and blood lactate levels in predicting NIMV failure. Our analysis revealed that the LAR (AUC=0.718, p<0.001) was a superior predictor of NIMV failure compared to serum albumin (AUC=0.603, p=0.066) and blood lactate levels (AUC=0.684, p=0.001) when considered independently. Furthermore, a cut-off value of 0.605 for LAR was identified, beyond which the likelihood of NIMV failure significantly increased (Figure 1) (Table 4).

**Table 4:** Comparison of diagnostic accuracy of lactate level, albumin level, and LAR for predicting NIMV failure *No significant cut-off value was identified for the serum albumin level in predicting the need for intubation. NIMV: noninvasive mechanical ventilation, LAR: lactate albumin ratio, PPV: positive predictive value, NPV: negative predictive value

	AUC (CI 95%)	Cut-off value	Sensitivity (%) (CI 95%)	Specificity (%) (CI 95%)	PPV (%) (CI 95%)	NPV (%) (CI 95%)	p-value
Lactate level	0.680 (0.578-0.791	≥2.1	53.49 (38.6-68.4%)	87.67 (80.1-95.2%)	71.88 (56.3-87.4%)	76.19 (67.0-85.4%)	0.001
Albumin level	0.603 (0.496-0.709)						0.066^*^
LAR	0.718 (0.616-0.819)	≥0.605	60.47 (45.9-75.1%)	84.93 (76.7-93.1%)	70.27 (55.6-85.0%)	78.48 (69.4-87.5%)	<0.001

The regression analysis of factors linked to NIMV failure revealed that an increase in the LAR corresponds to a 5.582-fold heightened risk of requiring intubation (OR: 5.582, 95% CI: 1.393-22.374, p=0.009). Additionally, other significant factors associated with increased intubation risk include elevated SOFA scores (OR: 1.342, 95% CI: 1.077-1.673, p=0.015) and higher pCO₂ levels (OR: 1.035, 95% CI: 1.007-1.064, p=0.014). While pH levels approached significance (p=0.050), APACHE-II scores, respiratory rate, and heart rate were not found to be significant predictors of intubation risk in this analysis (Table 5).

**Table 5:** Multivariate regression analysis of independent variables associated with NIMV failure NIMV: noninvasive mechanical ventilation, OR: odds ratio, CI: confidence interval, LAR: lactate albumin ratio, APACHE-II: Acute Physiology and Chronic Health Evaluation-II, SOFA: Sequential Organ Failure Assessment

	OR	95% CI	p-value
LAR	5.582	1.393-22.374	0.009
pH	0.200	0.04-1.001	0.050
APACHE-II score	1.116	0.943-1.321	0.203
SOFA score	1.342	1.077-1.673	0.015
pCO_2_	1.035	1.007-1.064	0.014
Respiratory rate	1.012	0.921-1.112	0.805
Heart rate	1.018	0.892-1.056	0.324

## Discussion

To the best of our knowledge, this is the first report on the role of LAR in predicting NIMV failure in patients with acute hypercapnic respiratory failure due to COPD exacerbation. Our findings demonstrate that the LAR serves as a significant predictor of NIMV failure, outperforming serum albumin and blood lactate levels in diagnostic accuracy. Given its ease of measurement and strong predictive capacity, the LAR could be integrated into current ICU risk stratification protocols alongside established scoring systems such as APACHE-II, SOFA, and HACOR. Incorporating LAR as an adjunct biomarker may enhance the early identification of patients at risk for NIMV failure, allowing clinicians to prioritize timely intubation or alternative supportive strategies. LAR could be particularly useful during the first 24 hours of ICU admission when rapid and dynamic assessment of a patient's metabolic status is critical. Additionally, patients who experienced NIMV failure exhibited more severe respiratory and metabolic derangements at the time of ICU admission, including higher SOFA scores and pCO₂ levels. These results suggest that early identification of patients at risk of NIMV failure, using LAR and other clinical parameters, could help optimize treatment strategies and improve patient outcomes.

Identifying predictors of NIMV failure has garnered significant interest due to its strong association with poor outcomes. The rate of NIMV failure can fluctuate significantly, ranging from 5% to 60%, depending on the specific cause of the respiratory failure. Therefore, predicting the risk of NIMV failure and closely monitoring the patient's clinical course during its application are critical to enhancing the success of the treatment and improving the patient's prognosis. The application of NIMV in hypercapnic ARF secondary to acute exacerbation of COPD, as well as its associated risk factors for failure, has been more extensively studied in the literature compared to its use in hypoxemic ARF [3,5,14]. 

NIMV failure can be predicted based on clinical and laboratory parameters, as well as disease severity and physical examination findings. Moreover, recent studies have shown that combining various clinical parameters can further enhance the accuracy of predicting NIMV failure in COPD patients [3,5,14-16]. Clinical studies indicate that a lower baseline pH is a significant risk factor for NIMV failure in patients with COPD. Specifically, NIMV failure has been observed in approximately 50-60% of patients presenting with an initial pH of less than 7.25 [17-19]. Additionally, higher pCO2 levels have been observed in patients who experience NIMV failure [20,21].

An initially high respiratory rate and its reduction after one hour of NIMV are linked to successful outcomes. Respiratory rates of 30-34 and ≥35 breaths/min at admission increase the risk of NIMV failure, with the odds further increasing if these elevated rates persist after two hours of NIMV [14,17,19,22,23]. Additionally, poor nutritional status, increased levels of white blood cell count, low serum potassium, and an increased heart rate are also risk factors for NIMV failure [24,25]. Consistent with previous clinical studies, in the present study, the NIMV failure group presented with a lower serum pH level (p=0.002), higher pCO₂ levels (p=0.011), elevated blood lactate levels (p=0.009), and lower blood albumin levels (p=0.029). These findings suggest that patients who experienced NIMV failure exhibited more pronounced metabolic and respiratory derangements upon ICU admission. However, there were no significant differences between the two groups in terms of respiratory rate (p=0.095).

Disease severity scores and the level of consciousness are considered important predictors of NIMV failure in cases of COPD-related acute hypercapnic respiratory failure. Specifically, elevated APACHE-II score, along with impaired consciousness, are key factors that increase the risk of NIMV failure in these patients [3,5,23,26]. In the study by Confalonieri et al., it was found that more than 70% of cases with a GCS <11, an APACHE-II score >28, a respiratory rate >29 breaths/min, and a pH <7.25 were associated with NIMV failure [19]. In the study by Antonio et al., the level of consciousness was reported as the only factor affecting NIMV success [27]. The HACOR score, a straightforward clinical metric, is frequently utilized to estimate the likelihood of NIMV success or failure, especially in ARF scenarios. A higher HACOR score (HACOR >5) is associated with an increased risk of NIMV failure, often indicating the need for closer monitoring or the potential need for invasive ventilation [14]. In the present study, it was determined that the GCS in the NIMV failure group was statistically significantly lower (p<0.001), suggesting a greater level of impaired consciousness in patients who failed NIMV. However, we found no statistically significant differences between the two groups in terms of APACHE-II and HACOR scores, indicating that these parameters may not be reliable predictors of NIMV failure in this patient population. In contrast, the SOFA score was significantly higher in the NIMV failure patient group, suggesting that a higher degree of organ dysfunction may be more closely associated with the risk of NIMV failure. This finding highlights the importance of monitoring SOFA scores for early identification of patients at higher risk of NIMV failure, allowing for timely interventions to improve outcomes.

Serum lactate level is one of the most commonly used biomarkers in the management of critically ill patients in both the emergency department and the ICU. Numerous studies have demonstrated a strong association between hyperlactatemia and poor survival outcomes in critically ill patients. Elevated lactate levels are commonly associated with hypoxia and hypoperfusion. Chronic hypoxia is a key characteristic of COPD, and under hypoxic conditions, increased glycolysis leads to a surge in lactate production. Additionally, hypoalbuminemia has been linked to poor prognosis and shorter survival times across various clinical conditions, further emphasizing its importance as a marker for patient outcomes. Therefore, lactate and albumin levels may be closely linked to outcomes in COPD patients [28-30]. In recent years, several studies have investigated the predictive value of the LAR for mortality across various clinical settings, addressing the limitations of relying solely on single measurements of lactate and albumin levels. The findings consistently show that an elevated LAR is strongly associated with increased mortality and organ dysfunction, particularly in patients with sepsis and septic shock. Moreover, these studies have demonstrated that LAR has superior performance in predicting mortality when compared to individual measurements of serum lactate or albumin levels, making it a more reliable prognostic marker in critically ill patients [7-12]. In our study, lower baseline pH, elevated pCO₂ levels, higher SOFA scores, and impaired consciousness (lower GCS scores) were all independently associated with NIMV failure. These findings align with previous literature demonstrating that severe respiratory acidosis, hypercapnia, and multisystem organ dysfunction at presentation are key predictors of poor NIMV outcomes. Consolidating these clinical and laboratory parameters may further enhance early risk stratification efforts.

The study by Xie J. et al., published in 2024, examines the predictive value of the LAR for 28-day mortality in patients admitted to the ICU with acute exacerbation of COPD. This retrospective cohort study evaluated patients diagnosed with acute exacerbation of COPD from the MIMIC-IV database. According to the results, the cutoff value of LAR was 0.645, as determined by ROC analysis. Kaplan-Meier analysis demonstrated that patients in the high LAR group (LAR >0.645) had a significantly higher 28-day mortality rate. A high LAR was found to be significantly associated with poor prognosis. This study indicates that an elevated LAR is associated with a significantly increased risk of 28-day mortality in acute exacerbation of COPD patients admitted to the ICU [28]. 

NIMV remains a cornerstone in the management of acute exacerbations of COPD, improving gas exchange and reducing intubation rates. Early identification of patients at high risk for NIMV failure is critical for optimizing outcomes. In this context, our study specifically explored the predictive capacity of LAR for NIMV failure, addressing an important gap in the literature.

The association between elevated LAR and NIMV failure may be explained by the pathophysiological impact of metabolic derangement and systemic compromise. Elevated lactate levels are indicative of tissue hypoxia, impaired oxygen utilization, and anaerobic metabolism, all of which suggest significant physiological stress. Concurrently, hypoalbuminemia reflects chronic inflammation, poor nutritional status, and diminished physiologic reserve. Together, a high LAR reflects both acute metabolic distress and impaired baseline resilience, predisposing patients to failure of noninvasive respiratory support due to an inability to meet the increased metabolic and respiratory demands.

Nonetheless, there remains a clear gap in the literature regarding whether LAR could be a reliable and early indicator of NIMV failure in patients with acute hypercapnic respiratory failure due to COPD exacerbation. Our study is the first to address this gap by investigating the prognostic value of LAR specifically in predicting NIMV failure in this patient population. This finding underscores the potential utility of LAR in clinical practice, facilitating the early detection of patients who may benefit from closer monitoring or an earlier switch to invasive mechanical ventilation.

This study has several limitations. First, it was conducted at a single tertiary care center, which may limit the generalizability of the findings to other institutions or populations. Second, the retrospective design inherently carries the risk of selection bias and missing data, and it prevents establishing a definitive cause-and-effect relationship. Third, the relatively small sample size may affect the statistical power and limit the ability to detect smaller but clinically significant differences. Additionally, certain potential confounding factors such as detailed nutritional status, long-term medication use, and specific comorbidities were not comprehensively evaluated. Finally, the lactate and albumin levels, and therefore the LAR, were measured only once within the first 24 hours of ICU admission; serial measurements could provide more insight into the dynamic nature of these biomarkers in relation to NIMV outcomes.

## Conclusions

LAR is a significant predictor of NIMV failure in COPD patients with acute hypercapnic respiratory failure. The superior predictive value of LAR compared to serum albumin and lactate levels indicates its potential utility in clinical practice for the early identification of patients at high risk for NIMV failure. This could allow for closer monitoring and timely interventions, such as the transition to invasive mechanical ventilation, improving patient outcomes in critical care settings. The findings underscore the potential of LAR as a comprehensive biomarker in guiding decision-making for the management of respiratory failure and lay the groundwork for future research in this area.
